# Application of Mesenchymal Stem Cells for Therapeutic Agent Delivery in Anti-tumor Treatment

**DOI:** 10.3389/fphar.2018.00259

**Published:** 2018-03-20

**Authors:** Daria S. Chulpanova, Kristina V. Kitaeva, Leysan G. Tazetdinova, Victoria James, Albert A. Rizvanov, Valeriya V. Solovyeva

**Affiliations:** ^1^OpenLab Gene and Cell Technologies, Institute of Fundamental Medicine and Biology, Kazan Federal University, Kazan, Russia; ^2^School of Veterinary Medicine and Science, University of Nottingham, Nottingham, United Kingdom

**Keywords:** mesenchymal stem cells, tumor microenvironment, membrane vesicles, cytokines, suppressor genes, oncolytic viruses, chemotherapy resistance

## Abstract

Mesenchymal stem cells (MSCs) are non-hematopoietic progenitor cells, which can be isolated from different types of tissues including bone marrow, adipose tissue, tooth pulp, and placenta/umbilical cord blood. There isolation from adult tissues circumvents the ethical concerns of working with embryonic or fetal stem cells, whilst still providing cells capable of differentiating into various cell lineages, such as adipocytes, osteocytes and chondrocytes. An important feature of MSCs is the low immunogenicity due to the lack of co-stimulatory molecules expression, meaning there is no need for immunosuppression during allogenic transplantation. The tropism of MSCs to damaged tissues and tumor sites makes them a promising vector for therapeutic agent delivery to tumors and metastatic niches. MSCs can be genetically modified by virus vectors to encode tumor suppressor genes, immunomodulating cytokines and their combinations, other therapeutic approaches include MSCs priming/loading with chemotherapeutic drugs or nanoparticles. MSCs derived membrane microvesicles (MVs), which play an important role in intercellular communication, are also considered as a new therapeutic agent and drug delivery vector. Recruited by the tumor, MSCs can exhibit both pro- and anti-oncogenic properties. In this regard, for the development of new methods for cancer therapy using MSCs, a deeper understanding of the molecular and cellular interactions between MSCs and the tumor microenvironment is necessary. In this review, we discuss MSC and tumor interaction mechanisms and review the new therapeutic strategies using MSCs and MSCs derived MVs for cancer treatment.

## Introduction

Due to their tropism to the tumor niche, mesenchymal stem cells (MSCs) are promising vectors for the delivery of antitumor agents. The isolation of MSCs from adult tissues poses circumvents many of the ethical and safety concerns which surround the use of embryonic or fetal stem cells, as these have been comprehensively discussed elsewhere (Herberts et al., 2011; Volarevic et al., 2018), this review focuses on the anti-tumor and therapeutic potential of MSCs. It is believed that the migration of MSCs toward the tumor is determined by inflammatory signaling similar to a chronic non-healing wound (Dvorak, 1986). It has been shown that MSCs are actively attracted to hepatic carcinoma (Xie et al., 2017), breast cancer (Ma et al., 2015), glioma (Smith et al., 2015) and pre-metastatic niches (Arvelo et al., 2016). However, the mechanism and factors responsible for the targeted tropism of MSCs to wounds and tumors microenvironments remain unclear. MSCs can migrate to sites of trauma and injury following the gradient of chemo-attractants in the extracellular matrix (ECM) and peripheral blood (Son et al., 2006) and local factors, such as hypoxia, cytokine environment and Toll-like receptors ligands, where upon arrival these local factors promote MSCs to express growth factors that accelerate tissue regeneration (Rustad and Gurtner, 2012).

It is believed, that following accumulation at the sites of tumor formation and growth, MSCs differentiate into pericytes or tumor-associated fibroblasts (TAF) thereby forming a growth supporting microenvironment and secreting such trophic factors as vascular endothelial growth factor (VEGF), interleukin 8 (IL-8), transforming growth factor β (TGF-β), epidermal growth factor (EGF), and platelet-derived growth factor (PDGF). (Nwabo Kamdje et al., 2017). For example, it has been shown that MSCs stimulate tumor growth and vascularization within the colorectal cancer xenograft model *in vivo* and can also induce activation of Akt and ERK in endothelial cells, thereby increasing their recruitment and angiogenic potential (Huang et al., 2013). Whilst in co-culture *in vitro* experiments, MSCs stimulated the invasion and proliferation of breast cancer cells (Pinilla et al., 2009).

However, besides tumor progression, MSCs can also supress tumor growth by cell cycle arrest and inhibition of proliferation, as well as blocking of PI3K/AKT pathway and tumor suppressor gene expression (Ramdasi et al., 2015). Anti-tumor properties are described for MSCs isolated from various sources in experiments both *in vitro* and *in vivo* of various tumor models (different tumor models are discussed in (Blatt et al., 2013a,b). For instance, MSCs injected into an *in vivo* model of Kaposi’s sarcoma suppressed tumor growth (Khakoo et al., 2006). Similar results have been reported for hepatoma (Qiao et al., 2008), pancreatic cancer (Cousin et al., 2009; Doi et al., 2010), prostate cancer (Chanda et al., 2009) and melanoma (Otsu et al., 2009) in both *in vitro* and *in vivo* models.

Thus, there are contradictory reports about the role of MSCs in tumor formation and development. The differences in the anticancer activity of MSCs reported by different group might be due to their activation status, which is discussed elsewhere (Rivera-Cruz et al., 2017). Nevertheless, there is a consensus that MSCs have enhanced tropism toward tumors which make them ideal vector candidates for targeted anti-tumor therapy.

## MSCs Migrate Toward Irradiated Tumors

Mesenchymal stem cells migration in the context of radiation therapy may also be very promising for cancer therapy. In fact, MSCs migrate better to irradiated 4T1 mouse mammary tumor cells in comparison to non-irradiated 4T1 cells (Klopp et al., 2007). Irradiated 4T1 cells are characterized by increased expression levels of TGF-β1, VEGF, and PDGF-BB. The activation of chemokine receptor CCR2 in MSCs interacting with irradiated 4T1 cells was also observed, as well as higher expression of MCP-1/CCL2 in the tumor parenchyma of 4T1 mice. Thus, MCP-1/CCL2/CCR2 signaling is important in the attraction of MSCs to irradiated tumor cells. Furthermore, CCR2 inhibition resulted in a significant decrease in MSC migration *in vitro* (Klopp et al., 2007). In irradiated glioma cells Kim et al. (2010) reported increased IL-8 expression, which led to an upregulation of IL-8 receptor by MSCs and an increase in their migration potential and tropism to glioma cells.

Once at the irradiated tumor site, MSCs can suppress immune cell activation directly through cell-cell interactions by binding the membrane protein PD-1 with PD-L1 and PD-L2 ligands on the T-lymphocyte surface. Moreover, MSCs can induce T-lymphocyte agonism by suppressing the expression of CD80 and CD86 on antigen-presenting cells (Yan et al., 2014a,b). Thus, the increased MSCs tropism to irradiated tumors may have the opposite effect in cancer therapy.

The described data clearly illustrate the correlation between tissue damage and MSCs recruitment. Due to an increase in tropism to the tumor, genetically modified MSCs can be an effective therapeutic tool. However, such therapeutic strategies can be risky for cancer patients since MSCs can potentially stimulate cancer progression within certain contexts.

## MSCs Chemotaxis Mediating Factors

Mesenchymal stem cells migrate to damaged tissue, trauma or sites of inflammation in response to secreted cytokines. Similarly, the tumor environment consists of a large number of immune cells, which alongside tumor cells, secrete soluble factors such as VEGF, PDGF, IL-8, IL-6, basic fibroblast growth factor (bFGF or FGF2), stromal cell-derived factor 1 (SDF-1), granulocyte colony-stimulating factor (G-CSF), granulocyte-macrophage colony stimulating factor (GM-CSF), monocyte chemoattractant protein 1 (MCP1), hepatocyte growth factor (HGF), TGF-β and urokinase-type plasminogen activator receptor (UPAR), attracting MSCs (Ponte et al., 2007).

Soluble factors CCL21 (Sasaki et al., 2008), IL-8 (Birnbaum et al., 2007), CXC3L1 (Sordi et al., 2005), IL-6 (Liu et al., 2011), macrophage inflammatory protein 1δ (MIP-1δ) and MIP-3α (Lejmi et al., 2015) directly mediate MSCs chemotaxis and recruitment to damaged tissues. IL-6 mediates chemotaxis, which facilitates MSC attraction into the main tumor growth sites (Rattigan et al., 2010). Ringe et al. (2007) observed the dose-dependent chemotactic activity of bone marrow-derived MSCs in relation to SDF-1α and IL-8. IL-8 dependent recruitment of MSCs was also detected in glioma. A multitude of angiogenic cytokines secreted by glioma cells, including IL-8, actively attract MSCs to tumor tissue (Ringe et al., 2007). Experiments with conditioned medium from Huh-7 hepatoma cell (Huh-7 CM) showed that MIP-1δ and MIP-3α induced MSC migration. Moreover, after cultivation of MSCs in Huh-7 CM the expression of matrix metalloproteinase 1 (MMP-1), necessary for migration, was significantly increased (Lejmi et al., 2015). It was also shown that PDGF-BB, VEGF and TGF-β1 can induce MSC migration (Schar et al., 2015). Experiments using MSCs modified with CXCR4, showed that increased expression of the CXCR4 receptor enhances MSC migration toward tumor cells in both *in vitro* and *in vivo* models (Kalimuthu et al., 2017). In osteosarcoma models, it was described that SDF-1α is involved in MSCs recruitment to tumor areas. MSCs in turn stimulate the migration of osteocarcinoma cells by CCL5/RANTES secretion (Xu et al., 2009), thereby promoting tumor invasion and metastatic colonization by providing metastatic osteosarcoma cells with a suitable microenvironment (Tsukamoto et al., 2012).

## Genetically Engineered MSCs With Anticancer Activity

In early studies MSCs genetically modified with interferon β (IFN-β) were injected into human melanoma mouse xenotransplantation models which resulted in decreased tumor growth and increased (2-times) survival of mice in comparison with controls (Studeny et al., 2002). In addition, it was shown in a melanoma xenograft mouse model that additional loading of IFN-β-modified canine MSCs with low amounts of cisplatin significantly increased the effectiveness of the antitumor therapy (Ahn et al., 2013).

Currently, besides IFN-β there are several other cytokines and tumor-suppressor genes with anticancer activity which are used for genetic modification of MSCs (**Table 1**). One of the most promising therapeutic pro-apoptotic cytokines is tumor necrosis factor (TNF)-related apoptosis-inducing ligand (TRAIL), which selectively induces apoptosis in cancer cells. The antitumor effect of TRAIL-modified MSCs has been described for different types of tumors, within which TRAIL has not been found to be cytotoxic for normal mammalian cells and tissues (Szegezdi et al., 2009; Yuan et al., 2015). It is interesting that recombinant TNF-α-activated MSCs in combination with radiation exposure are able to significantly increase expression level of endogenous TRAIL (Mohammadpour et al., 2016). Long-lasting expression of endogenous TRAIL can also be observed in IFN-γ-modified MSCs (Yang X. et al., 2014). To increase the therapeutic potential of TRAIL-modified MSCs, it has been suggested they could be used in combination with chemotherapeutic agents, such as cisplatin (Zhang et al., 2012). However, some tumors have mechanism of TRAIL resistance through overexpression of X-linked inhibitory of apoptosis protein (XIAP), which inhibits caspase 3 and 9 activation. Anti-apoptotic properties of XIAP are under control of the second mitochondria-derived activator of caspase (Smac), which prevents physical interaction of XIAP and caspases thereby preventing apoptosis inhibition (Srinivasula et al., 2001). Khorashadizadeh et al. (2015) used MSCs for the delivery and simultaneous expression of novel cell penetrable forms of Smac and TRAIL. The effectiveness of this approach was shown in TRAIL-resistant breast cancer cell line MCF-7 (Khorashadizadeh et al., 2015).

**Table 1:** The usage of genetically engineered Mesenchymal stem cells for target delivery of therapeutic agents with anti-tumor activity.

Agent	Mechanism of action	Model	Reference
IFN-α	Immunostimulation, apoptosis induction, angiogenesis suppression	Immunocompetent mouse model of metastatic melanoma	Ren et al., 2008a
IFN-β	Increased activity of NK cells, inhibition of	Mouse 4T1 breast tumor model	Ling et al., 2010
	Stat3 signaling	Mouse prostate cancer lung metastasis model	Ren et al., 2008b
		PC-3 (prostate cancer) xenograft model	Wang et al., 2012
		PANC-1 (pancreatic carcinoma) xenograft model	Kidd et al., 2010
IFN-γ	Immunostimulation, apoptosis induction	*In vitro* human leukemia cell line K562	Li et al., 2006
TRAIL	Caspase activation, apoptosis induction	Orthotopic model of Ewing sarcoma	Guiho et al., 2016
		Subcutaneous model of lung cancer	Mohr et al., 2008; Yan et al., 2016
		Xenograft model of human malignant mesothelioma	Sage et al., 2014; Lathrop et al., 2015
		Colo205 (colon cancer) xenograft tumor model	Marini et al., 2017
		Xenograft model of human myeloma	Cafforio et al., 2017
		Xenograft model of human tongue squamous cell carcinoma (TSCC)	Xia et al., 2015
		Eca-109 (esophageal cancer) xenograft model	Li et al., 2014
		Xenograft model of human glioma	Kim et al., 2010; Choi et al., 2011; Wang et al., 2017
IL-2	Immunostimulation	Rat glioma model	Nakamura et al., 2004
IL-12	Immune system cell activation	Liver cancer H22 and MethA ascites models	Han et al., 2014
		Mouse model bearing subcutaneous SKOV3 (ovarian carcinoma) tumor explants	Zhao et al., 2011
		Xenograft model of human glioma	Hong et al., 2009; Ryu et al., 2011
IL-21	Immunostimulation	Mouse model of B-cell lymphoma	Kim et al., 2015
		A2780 (ovarian cancer) xenograft model	Hu et al., 2011
PTEN	Induction of G(1)-phase cell cycle arrest	*In vitro* glioma cell line	Yang Z.S. et al., 2014; Guo et al., 2016
CX3CL1	Cytotoxic T cells and NK cells activation	Mice bearing lung metastases of C26 (colon carcinoma) and B16F10 (skin melanoma) cells	Xin et al., 2007
HSV-TK/GCV	Drug precursors transformation	9L (glioma) xenograft model	Uchibori et al., 2009
		*In vitro* glioma cell lines 8-MG-BA, 42-MG-BA and U-118 MG	Matuskova et al., 2010
CD/5-FC	Drug precursors transformation	Subcutaneous model of melanoma or colon cancer	Kucerova et al., 2007, 2008
		Cal72 (osteosarcoma) xenograft model	NguyenThai et al., 2015
NK4	Apoptosis induction, angiogenesis and	C-26 lung metastasis model	Kanehira et al., 2007
	lymphangiogenesis suppression	Nude mice bearing gastric cancer xenografts	Zhu et al., 2014
		MHCC-97H (liver carcinoma) xenograft model	Cai et al., 2017
Oncolytic viruses	Tumor destruction by virus replication	Orthotopic breast and lung tumors	Hakkarainen et al., 2007
		Mouse glioblastoma multiforme models	Duebgen et al., 2014
		A375N (melanoma) tumor xenografts	Bolontrade et al., 2012
PEDF	Inhibiting tumor angiogenesis, inducing apoptosis,	Lewis lung carcinoma (LLC) xenograft model	Chen et al., 2012
	and restoring the VEGF-A/sFLT-1 ratio	Mice bearing U87 gliomas	Su et al., 2013
		CT26 CRPC model	Yang et al., 2016
Apoptin	Tumor destruction, caspase 3 activation	HepG2 (hepatocellular carcinoma) tumor xenografts	Zhang et al., 2016
		Lung carcinoma xenograft model	Du et al., 2015
HNF4-α	Wnt/β-catenin pathway inhibition	SK-Hep-1 (hepatocellular carcinoma) tumor xenografts	Wu et al., 2016
miR-124	Increase the differentiation of glioma stem cells	Glioma tumor cells in a spheroid cell culture system	Lee et al., 2013
	by targeting SCP-1 or CDK6	*In vitro* human glioblastoma multiforme cell line	Sharif et al., 2017
miR-145	Sox2 and Oct4 expression inhibition	Glioma tumor cells in a spheroid cell culture system	Lee et al., 2013

Besides IFN-β and TRAIL as anti-tumor agents, interleukins are also under consideration because they regulate inflammation and immune responses For instance, IL-12-modified MSCs decrease metastasis and induce cancer cell apoptosis in mice with melanoma, lung cancer and hepatoma by 75, 83, and 91%, respectively. The activation of immune cells [cytotoxic T-lymphocytes and natural killers (NK)] was also reported (Chen et al., 2008). You et al. (2015) showed that injection of genetically modified amniotic fluid-derived MSCs expressing IL-2 resulted in induction of apoptosis in ovarian cancer cells in an *in vivo* mouse model.

PTEN (phosphatase and tensin homolog deleted on chromosome 10) is one of the main human tumor-suppressors. Yang Z.S. et al. (2014) showed that PTEN expressing MSCs are able to migrate toward DBTRG (brain glioblastoma) tumor cells *in vitro*. PTEN-modified MSCs anti-cancer activity in co-culture with U251 glioma cells *in vitro* was also described (Guo et al., 2016). MSC-mediated delivery and anti-tumor properties were described for other proteins (IFN-α, IFN-γ, CX3CL1, apoptin, PEDF) and ncRNAs (miR-124 and miR-145) (**Table 1**). Modification of MSCs for the co-expression of several therapeutic proteins can increase their anti-cancer potential. It was shown that TRAIL and herpes simplex virus thymidine kinase (HSV-TK) modified MSCs in the presence of ganciclovir (GCV) significantly reduced tumor growth and increased survival of mice with highly malignant glioblastoma multiform (GBM) (Martinez-Quintanilla et al., 2013).

The effect of direct administration of many of these agents in cancer treatment is often limited due to their short half-life in the body and pronounced toxicity in relation to normal, non-cancerous cells. The use of MSCs for delivery of the above mentioned therapeutic proteins can help to minimize such problems because MSCs can selectively migrate to tumor sites and exert therapeutic effects locally thereby significantly increasing the concentration of the agent in the tumor and reducing its systemic toxicity.

Another promising approach is delivery of oncolytic viruses with MSCs. For instance, Du et al. (2017) used MSCs as a vector for the delivery of oncolytic herpes simplex virus (oHSV) [approved by Food and Drug Administration (FDA) for melanoma treatment] in human brain melanoma metastasis models in immunodeficient and immunocompetent mice. Authors noted that the introduced MSCs-oHSV migrated to the site of tumor formation and significantly prolonged the survival of mice. In the immunocompetent model a combination of MSCs-oHSV and PD-L1 blockade increases IFNγ-producing CD8^+^ tumor-infiltrating T lymphocytes and results in a significant increase of the median survival of treated animals (Du et al., 2017).

## MSCs Primed With Anticancer Drugs

Mesenchymal stem cells relative resistance to cytostatic and cytotoxic chemotherapeutic drugs and migration ability opens new ways to use them for targeted delivery of therapeutic drugs directly to tumor sites. Pessina et al. (1999) showed that SR4987 BDF/1 mouse bone marrow stromal cells can be a reservoir for doxorubicin (DOX) which can subsequently be released not only in the form of DOX metabolites but also in its original form. It was further shown that MSCs efficiently absorb and release paclitaxel (PTX) in an active form (Pascucci et al., 2014), DOX, and gemcitabine (GCB), all having an inhibitory effect on tongue squamous cell carcinoma (SCC154) cells growth *in vitro* (Cocce et al., 2017b).

Pessina et al. (2013) found that the maximum concentration of PTX which did not affect MSC viability was 10 000 ng/mL. The concentration is sufficient to decrease the viability of certain types of tumor cells, for example, human leukemia cells. *In vivo* investigations show that PTX-primed MSCs (MSCs-PTX) demonstrate strong antitumor activity inhibiting the growth of tumor cells and vascularization of the tumor in a MOLT-4 (leukemia) xenograft mouse model (Pessina et al., 2013). The anti-tumor activity of primed MSCs is currently being investigated on the different types of tumor cells. For instance, Bonomi et al. (2017b) showed that MSCs-PTX suppress the proliferation of human myeloma cells RPMI 8226 in *in vitro* 3D dynamic culture system. The anti-cancer activity of MSCs-PTX has been further shown in relation to pancreatic carcinoma cells *in vitro* (Brini et al., 2016).

Nicolay et al. (2016) showed that cisplatin (CDDP) had no significant effect on cell morphology, adhesion or induction of apoptosis in MSCs, nor does it affect their immunophenotype or differentiation potential of MSCs once primed with CDDP. This has been confirmed using CDDP at concentrations of 2.5 μg/ml and 5.0 μg/ml (Gilazieva et al., 2016). Thus, MSCs are promising vectors for CDDP delivery toward the tumor sites.

Beside chemical drugs in soluble form, MSCs can absorb nanomaterials containing chemotherapeutic agents. For instance, MSCs primed with silica nanoparticle-encapsulated DOX promoted a significant increase in the apoptosis of U251 glioma cells *in vivo* (Li et al., 2011).

Bonomi et al. (2017a) in their work used MSCs from two sources: dog adipose tissue and bone marrow, to study MSCs-PTX antitumor activity on human glioma cells (T98G and U87MG). The investigation once again showed the pronounced antitumor effect of MSCs-PTX and opens new perspectives for oncological disease therapy not only in humans but also in animals (Bonomi et al., 2017a).

## MSC-Derived Microvesicles

Extracellular vesicles (EVs) [microvesicles (MVs) and exosomes] released by a large number of cells play an important role in intercellular communication. MVs from different cell types contain biologically active functional proteins, and nucleic acids including mRNA and microRNA (Pokharel et al., 2016). It was shown that MSC-derived MVs can promote progression of various types of tumors. For instance, MSC-derived MVs have been found to facilitate the migration of MCF7 breast cancer cells by activating the Wnt signaling pathway (Lin et al., 2013), promote the progression of nasopharyngeal carcinoma cells (Shi et al., 2016) and increase the proliferation and metastatic potential of gastric cancer cells (Gu et al., 2016). MSC-derived MVs can also increase tumor cell resistance to drugs. For example, MSC-derived MVs can induce resistance to 5-fluorouracil in gastric cancer cells by activating the CaM-Ks/Raf/MEK/ERK pathway (Ji et al., 2015). Bliss et al. (2016) showed that a possible cause of increased resistance to chemotherapy are micro-RNAs which are included in MVs, such as miR-222/223, which support the resistance of the breast cancer cells in the bone marrow. However, there are conflicting results, for example Del Fattore et al. (2015) reported that MVs isolated from bone marrow and cord blood-derived MSCs suppressed division and induced apoptosis in glioblastoma cells. However, MVs isolated from adipose tissue-derived MSCs showed the opposite effect and stimulated tumor cell proliferation (Del Fattore et al., 2015). As mentioned above, such differences might be explained by activation status of parental MSCs from which the MVs are generated.

One of the possible approaches to use MSCs-isolated MVs in therapy is via the priming/loading of these structures with therapeutic agents. Pascucci et al. (2014) demonstrated that the antitumor activity of MSCs-PTX may be due to the release of a large number of MVs by the MScs. Loaded with PTX MSCs demonstrate vacuole-like structures and accumulation of MVs in extracellular space without significant change in cell morphology. Presence of PTX in MVs was confirmed by Fourier spectroscopy. The release of PTX containing MVs were found to exert anti-cancer activity which was confirmed using the human pancreatic adenocarcinoma cell line CFPAC-1 *in vitro* (Pascucci et al., 2014). This finding was supported by the recent studies of Cocce et al. (2017a) which showed antitumor activity of MVs derived from MSCs-PTX and MSCs-GCB on pancreatic cancer cells *in vitro*.

Yuan et al. (2017) investigated antitumor activity of MSC-derived MVs carrying recombinant TRAIL (rTRAIL) on their surface. Cultivation of M231 breast cancer cells in the presence of MVs led to the induction of apoptosis in cancer cells. At the same time, MVs did not induce apoptosis in normal human bronchial epithelial cells (HBECs). The use of MSC-derived MVs bearing rTRAIL on their surface proved to be more effective than using pure rTRAIL (Yuan et al., 2017).

Kalimuthu et al. (2016) developed bioluminescent EVs using *Renilla luciferase* (Rluc)-expressing MSCs (EV-MSC/Rluc) and showed that these vesicles migrate at tumor sites in the Lewis lung carcinoma (LLC) model *in vivo*. Significant cytotoxic effect of EV-MSC/Rluc on LLC and 4T1 cells *in vitro* was also noticed. Moreover, EV-MSC/Rluc inhibited LLC tumor growth *in vivo* (Kalimuthu et al., 2016).

## Conclusion

**FIGURE 1:**
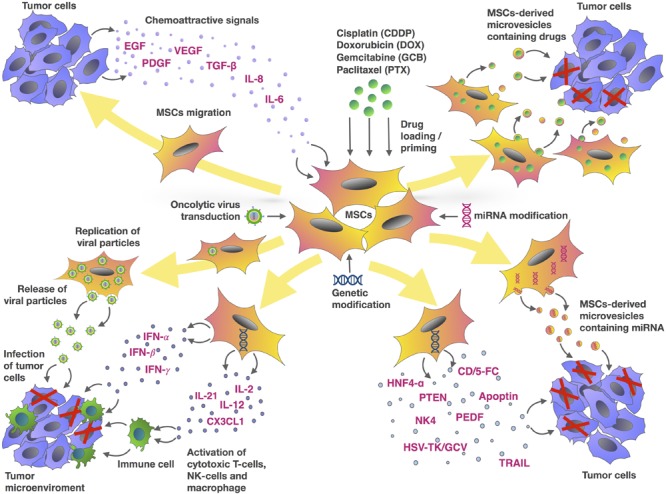
Mesenchymal stem cells and tumor cells interaction as an MSC-based approach for cancer therapy. The chemotactic movement of MSCs toward a tumor niche is driven by soluble factors, such as VEGF, PDGF, IL-8, IL-6, bFGF or FGF2, SDF-1, G-CSF, GM-CSF, MCP1, HGF, TGF-β, and UPAR. Genetic modification of MSCs can be used to deliver a range of tumor-suppressing cargos directly into the tumor niche. These cargos include tumor suppressor (TRAIL, PTEN, HSV-TK/GCV, CD/5-FC, NK4, PEDF, apoptin, HNF4-α), oncolytic viruses, immune-modulating agents (IFN-α, IFN-γ, IL-2, IL-12, IL-21, IFN-β, CX3CL1), and regulators of gene expression (miRNAs and other non-coding RNAs). MSCs are also capable of delivering therapeutic drugs such as DOX, PTX, GCB, and CDDP within the tumor site. In addition to using MSCs directly, microvesicles (MVs) isolated from MSCs represent an alternative approach to delivering these agents.

Tumor development and response to therapy depends not only on tumor cells, but also on different cell types which form the stroma and microenvironment. These include immune cells, vascular endothelial cells and tumor-associated stromal cells such as TAF and MSCs. Due to tropism to the tumor microenvironment, MSCs can be considered as promising vectors for the delivery of antitumor agents (**Figure 1**). To date, there are large number of experimental studies that confirm the anti-oncogenic potential of MSCs modified with therapeutic genes and/or loaded with chemotherapeutic drugs. Thus, the approach of therapeutic agent delivery to the tumor sites using MSCs is promising. However, since it is known that native MSCs can exhibit not only anticancer but also pro-oncogenic properties, further research is needed to improve the safety of this approach. An alternative to using intact MSCs to deliver anti-tumor agents, is the use of MSC-derived MVs which can also be loaded with the same antitumor agents. Further research is needed to evaluate the safety and efficiency of the different therapeutic approaches described in this review to harness the promising potential of MSCs as therapeutic vectors.

## Author Contributions

DC wrote the manuscript and made the table. KK and VJ collected the data of homing of MSCs. LT collected the information of MSCs priming. KK made the figure. DC, VS, and AR conceived the idea and edited the manuscript, figure, and table.

## Conflict of Interest Statement

The authors declare that the research was conducted in the absence of any commercial or financial relationships that could be construed as a potential conflict of interest.
